# The complete mitochondrial genome of *Triplax ainonia* Lewis, 1877 (Coleoptera: Erotylidae)

**DOI:** 10.1080/23802359.2023.2179358

**Published:** 2023-02-20

**Authors:** Ben Hong, Yunxue Xiao, Changqing Luo

**Affiliations:** a Institute of Entomology, The Provincial Key Laboratory for Agricultural Pest Management Mountainous Region, Guizhou University, Guiyang, China; b Xishuangbanna Tropical Botanical Garden, Chinese Academy of Sciences, Mengla, China

**Keywords:** Beetles, mitochondrial genome, phylogeny, Erotylidae, mushrooms

## Abstract

The beetle *Triplax ainonia* Lewis, 1877 is a serious pest of cultivated the mushroom *Pleurotus ostreatus* in China. The complete mitochondrial genome of this species was reported for the first time in this study. The mitogenome was 17,555 bp in length and had a base composition of 39.4% A, 36.1% T, 8.7% G and 15.3% C, which indicated that the base composition was AT-biased. Similar to other species of Coleoptera, the mitogenome of *T. ainonia* contained 13 protein-coding genes, 22 transfer RNA genes, two ribosomal RNA unit genes, and a large noncoding region. Phylogenetic analysis based on mitogenomes suggested that the family Erotylidae was a monophyletic group.

## Introduction

The genus *Triplax* Fabricius belongs to the tribe Tritomini of the subfamily Erotylinae, and the species of this genus are widely distributed in East Asia such as China, Korea and Japan (Jung and Park 2017; Jung 2019). Most species of the genus *Triplax* are fungivorous (Goodrich and Skelley 1993; Jung 2019). For example, we found that both the adults and the larvae of the species *Triplax ainonia* Lewis, 1877 can feed on the fruit bodies of the *Pleurotus ostreatus* and become a serious pest of this cultivated mushroom in China. This beetle species has the following typical morphological features: body elongate oval, mostly shiny and black; body length 2.82–4.86 mm, and width 1.63–2.67 mm; elytra bright black, head black; pronotum with two circular and black markings; antennae brown, but 9–11antennomeres dark brown; legs and abdomen orange. In this study, we report the complete mitochondrial genome of *T. ainonia*, representing the first mitogenome of the genus *Triplax*, and the results of this study will contribute to the species identification of this pest based on molecular data.

## Materials and methods

**Figure 1.**
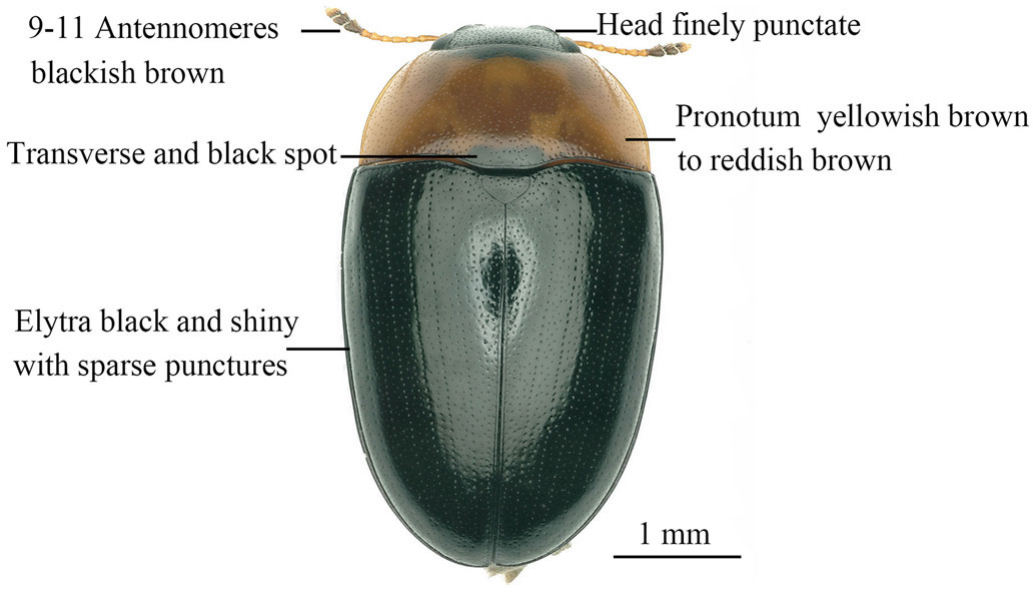
Adult habitus of *Triplax ainonia* Lewis, 1877 (this photo was taken by Ben Hong, the first author of this article).

The specimens of the *Triplax ainonia* were collected from Baijin Town, Huishui County, South Guizhou Autonomous Prefecture, Guizhou Province, China (26°6′32″N, 106°50′9″E) on 16 November 2021. The insect specimens used in this study were collected and identified by the author (Ben Hong). A specimen was deposited at the Institute of Entomology, Guizhou University (http://www.gzu.edu.cn/, Changqing Luo, luochangqing13@163.com) under the voucher number GZU IE2021100101. The sample was preserved in 100% ethanol at −20 °C (Figure 1).

Genomic DNA of *T. ainonia* was extracted from one leg using the CTAB method (Reineke et al. 1998). DNA samples were stored at the Institute of Entomology, Guizhou University (http://www.gzu.edu.cn/, Changqing Luo, luochangqing13@163.com) under the voucher number GZU E2021112801. A total amount of 1 µg DNA per sample was used as input material for the DNA library preparations. The DNA libraries were sequenced on Illumina NovaSeq 6000 platform (Illumina, USA), and 150 bp paired-end (PE) reads were generated. After quality assessment, a total of 38,710,978 raw reads were used to assemble the mitochondrial genome, and the assembly was annotated and visualized using MitoZ (Meng et al. 2019). Circularity was checked using the MITOS Web Server (Bernt et al. 2013, http://mitos.bioinf.uni-leipzig.de/), and manually revised based on the mitogenome of the species *Neotriplax arisana* which is closely related to *T. ainonia*. Thirteen protein-coding genes (PCGs) of the mitogenomes of five species, including the mitogenome of the species *T. ainonia* and all other known mitogenomes of the species belonging to the family Erotylidae of the Cucujoidea, and the mitogenomes of the species of four families (each family with two representative species) which also belong to the superfamily Cucujoidea were used for the phylogenetic analysis. One species of another superfamily (i.e. Sphaerioidea) of the Coleoptera was employed as the outgroup taxon. Except for the species *T. ainonia*, the complete mitochondrial sequences of the other 13 beetle species were downloaded from the NCBI (www.ncbi.nlm.nih.gov). Multiple sequence alignment was carried out using Clustal X 1.81 (Thompson et al. 1997). The maximum likelihood tree was reconstructed by MEGA X (Kumar et al. 2018) with 1000 bootstrap replications. The best model of evolution for the ML analyses was selected with MEGA X (Kumar et al. 2018), and the GTR + G+I was chosen as the final model of nucleotide substitutions.

## Results

**Figure 2.**
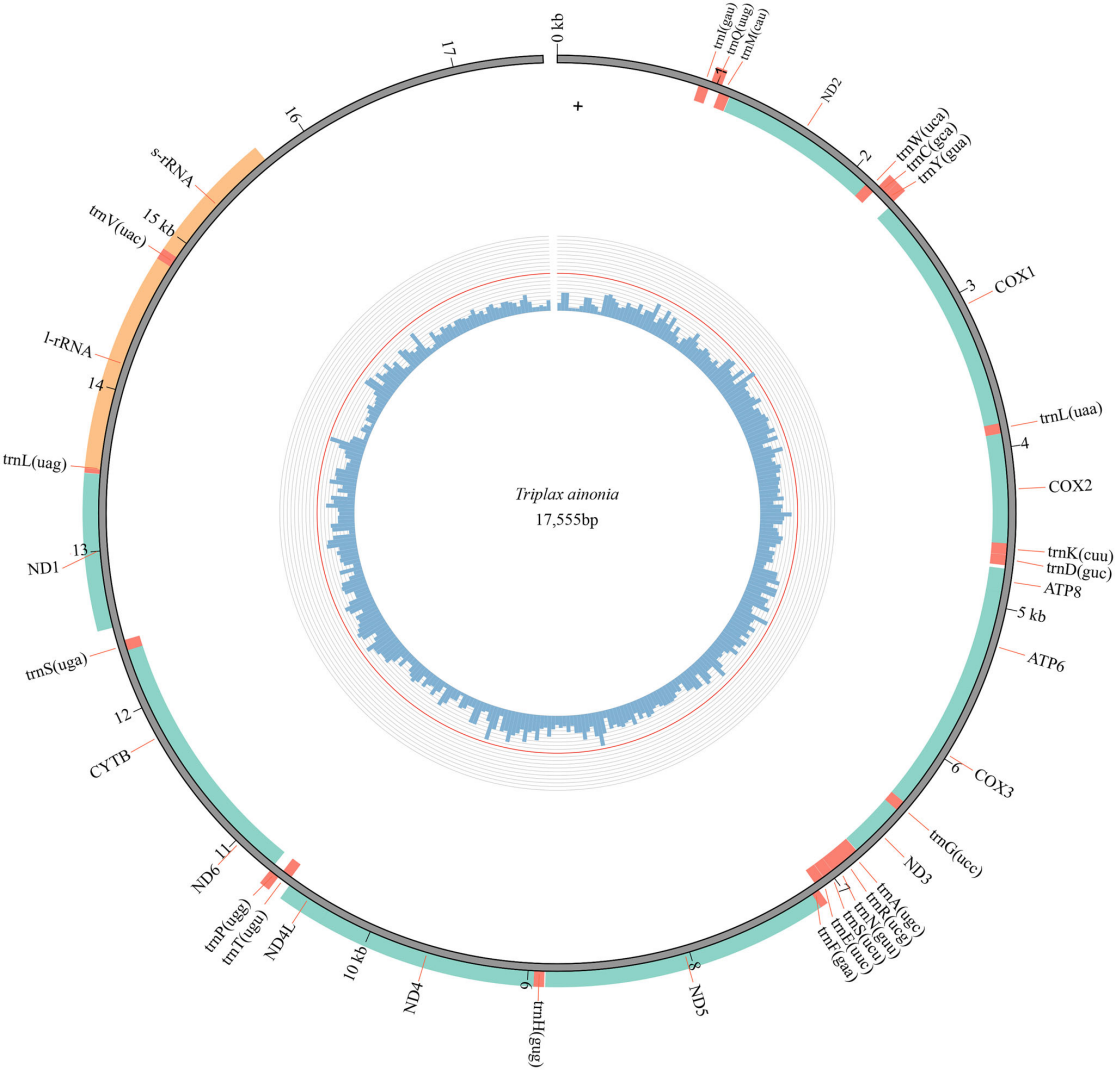
Genome map of the mitogenome of *T. ainonia*. The outermost circle shows the arrangement of the genes: blue for the CDS, red for tRNAs, and orange for rRNAs.

The complete mitochondrial genome of *Triplax ainonia* is a typical circular double-strand DNA molecule of 17,555 bp (GenBank accession no. OM084946). The mitogenome of this species contains 37 genes: 13 protein-coding genes (PCGs), 22 transfer RNA genes (tRNAs), the small and large ribosomal RNA unit genes (*rrnS* and *rrnL*), and a large noncoding region (putative control region) (Figure 2). The mitogenome of *T. ainonia* has a base composition of 39.4% A, 36.1% T, 8.7% G, 15.3% C.

Twelve protein-coding genes use ATN (ATG, ATT, and ATA) as the start codons. Among those genes, six PCGs (*cox2*, *cox3*, *nad4*, *nad6*, *cob*, and *atp6*) start with ATG, five PCGs (*cox1*, *atp8*, *nad3*, *nad5*, and *nad4l*) start with ATT, and one PCG (*nad2*) starts with ATA. For the protein-coding gene *nad1*, TTG is used as a start codon. Eleven protein-coding genes stops with TAA, and the gene *nad5* uses the TAG as a stop codon. However, the gene *nad4* stop with an incomplete stop codon TA. The large noncoding region is 2,742 bp long, and located between *rrnS* and *trnl*. The 22 tRNAs are interspersed among ribosomal RNAs and protein-coding regions, and the size of these tRNAs varies from 64 bp (*trnC*, *trnH*, and *trnT*) to 70 bp (*trnK* and *trnV*). The *rrnS* is located between *trnV* and the noncoding region, and the *rrnL* is located between *trnL* and *trnV*. The *rrnS* and *rrnL* are 811 bp and 1329 bp long, respectively.

**Figure 3.**
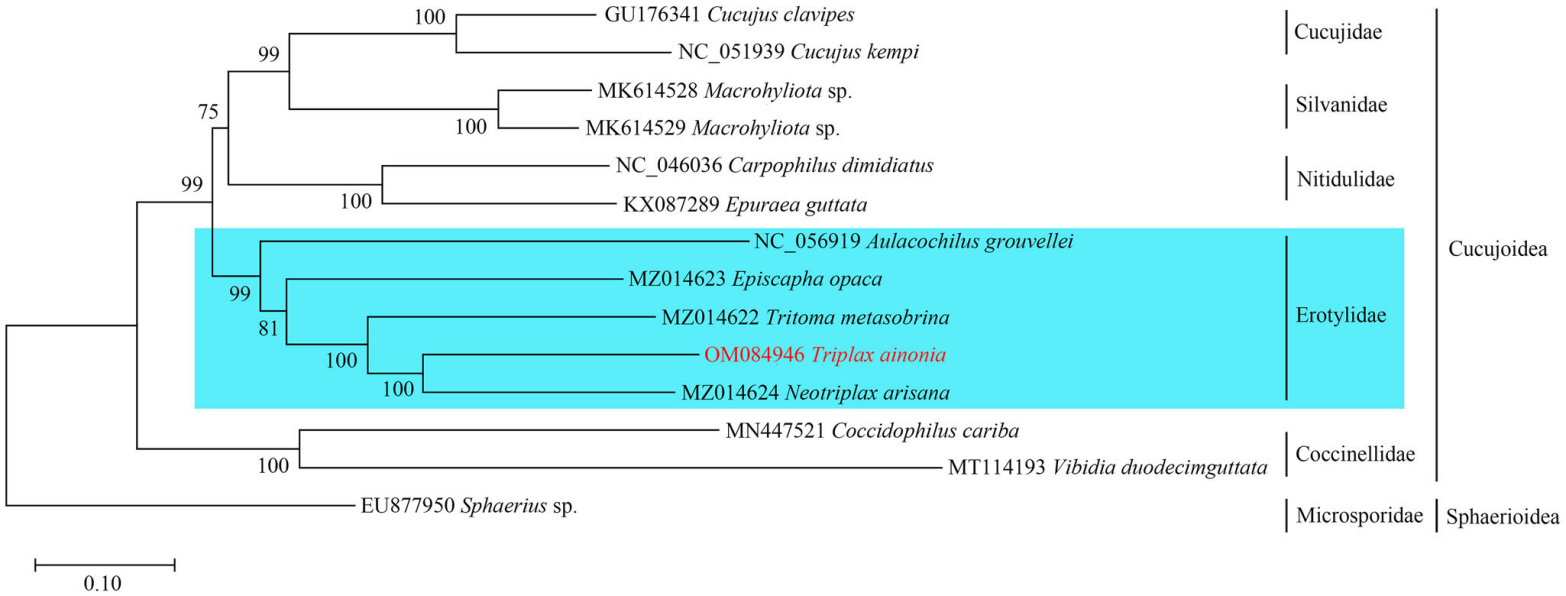
The phylogenetic tree resulting from maximum likelihood analysis. Numbers at the nodes represent bootstrap percentages. The following sequences were used: *Cucujus clavipes* GU176341 (Song et al. 2010); *Cucujus kempi* NC_051939, *Macrophyliota* sp. MK614528, *Macrophyliota* sp. MK614529 (Jin et al. 2020); *Carpophilus dimidiatus* NC_046036, *Epuraea guttata* KX087289 (Chen et al. 2020); *Aulacochilus grouvellei* NC_056919 (Liu et al. 2021); *Episcapha opaca* MZ014623, *Tritoma metasobrina* MZ014622, *Neotriplax arisana* MZ014624 (Liu et al. 2021); *Coccidophilus cariba* MN447521 (Nattier and Salazar 2019); *Vibidia duoecimguttata* MT114193 (Yan et al. 2020); *Sphaerius* sp. EU877950 (Sheffield et al. 2008).

Phylogenetic relationships for 13 beetle species belonging to five families of the Cucujoidea were reconstructed (Figure 3). The inferred phylogeny tree showed that the family Erotylidae which comprises the beetle species *T. ainonia* was a monophyletic group with 99% bootstrap probability (Figure 3). Furthermore, the other four families formed a well-supported monophyletic clade, respectively (BP = 100%) (Figure 3).

## Discussion and conclusions

This study determined the main features of the mitochondrial genome of the beetle *Triplax ainonia*. The gene composition of the mitogenome of this species resembles the mitogenomes of other beetle species belonging to Coleoptera (Liu et al. 2021; Xing et al. 2022). In addition, similar to the mtDNA sequences of other insects, the base composition of *T. ainonia* mitogenome is biased toward A + T bases (Yang et al. 2009). In the beetle *T. ainonia*, the gene *nad4* stops with an incomplete stop codon, and this phenomenon of mitochondrial genes with immature stop codons have also been reported in other beetles (Liu et al. 2021; Xing et al. 2022).

The phylogenetic analysis showed that the Erotylidae formed a monophyletic clade, and the monophyly of this family was also confirmed by previous phylogenetic studies based on morphological and molecular characters (Lawrence and Newton 1982; Leschen et al. 2005; McElrath et al. 2015; Liu et al. 2021). In our results, the family Erotylidae was the sister to the other three families: Cucujidae + Silvanidae + Nitidulidae, and this topology was strongly supported. However, clarification of the phylogenetic relationships among families of the Cucujoidea need further phylogenetic studies which should be based on a much broader taxon sampling of this superfamily.

## Ethical approval

The study was approved by the Guizhou University Subcommittee of Experimental Animal Ethics (approval no. EAE-GZU-2021-E015).

## Data Availability

The mitochondrial genome sequence data that support the findings of this study are openly available in GenBank of NCBI at (https://www.ncbi.nlm.nih.gov/) under the accession no. OM084946. The associated BioProject, SRA, and Bio-Sample numbers are PRJNA798621, SRR17731626, and SAMN25118147, respectively.
